# Development and Performance Verification of the PBPK Model for Antazoline and Its Metabolite and Its Utilization for Pharmacological Hypotheses Formulating

**DOI:** 10.3390/ph15030379

**Published:** 2022-03-20

**Authors:** Barbara Wiśniowska, Joanna Giebułtowicz, Roman Piotrowski, Piotr Kułakowski, Sebastian Polak

**Affiliations:** 1 Department of Social Pharmacy, Faculty of Pharmacy, Jagiellonian University Medical College, Medyczna 9, 30-688 Kraków, Poland; sebastian.polak@uj.edu.pl; 2 Department of Bioanalysis and Drugs Analysis, Faculty of Pharmacy, Medical University of Warsaw, Banacha 1, 02-097 Warsaw, Poland; joanna.giebultowicz@wum.edu.pl; 3 Department of Cardiology, Centre of Postgraduate Medical Education, Grochowski Hospital, 04-073 Warsaw, Poland; rpiotrow@op.pl (R.P.); piotr.kulakowskimd@gmail.com (P.K.); 4 Simcyp Division, Certara UK Limited, Level 2-Acero, 1 Concourse Way, Sheffield S1 2BJ, UK

**Keywords:** atrial fibrillation, antazoline, PBPK modelling, IVIVE

## Abstract

Antazoline is an antihistaminic drug that is effective in the termination of paroxysmal atrial fibrillation. Despite its long presence in the market, antazoline’s ADME parameters and pharmacokinetic effects in humans are poorly characterized. The objective of this study was to fill this gap by generation of in vitro and in vivo data and the development of a physiologically based pharmacokinetic model describing antazoline and its main metabolite disposition. A set of ADME parameters for the antazoline and its hydroxy metabolite is provided based on literature data, QSAR predictions, in vitro binding and metabolic stability assays. These can be used to feed PBPK models. In our current work, the developed PBPK model simulating simultaneously the pharmacokinetic profile of antazoline and its metabolite was successfully verified against the available clinical data and the presented capability to account for the clinically observed variability. When used to feed the PD model (e.g., simulating ECG), concentration-time profiles predicted by the model enable the assessment of antazoline’s effect in various clinical scenarios with the possibility to account for population differences or CP mediated drug-drug interactions.

## 1. Introduction

### 1.1. Antazoline

Antazoline is a first-generation antihistaminic drug that has now been marketed for over 50 years. Currently, its use for the treatment of allergy symptoms is very limited due to poor receptor selectivity and its side effects, mostly in terms of the central nervous system. However, during the last decade, new data have emerged showing that antazoline also has an antiarrhythmic activity with quinidine-like properties and is effective in the termination of paroxysmal atrial fibrillation (AF) [1,2,3,4]. In addition to the fact that antazoline quickly and effectively terminates AF, it is also safe in the elderly and in the patients with stable coronary artery disease [5,6]. These effects of antazoline’s use in cardiology and emergency medicine have been recently described in a comprehensive review published by [7]. The drug lowers the velocity of intra-atrial conduction, prolongs the atrial refraction period, and improves atrioventricular conduction, allowing a fast ventricular response to supraventricular arrhythmias [8]. Although the detailed mechanisms of antiarrhythmic action at the cellular and sub-cellular levels remain unknown, the inhibition of ion currents can be expected to play a role. Recently published results of a clinical study on antazoline with PK and PD endpoints [9] demonstrated that antazoline prolonged conduction (prolongation of the P wave and increase of QRS complex duration) and repolarization (prolongation of QT/QT corrected for the heart rate using Fridericia formula-QTcF interval) [1].

Information about ADME parameters and PK human data describing antazoline’s disposition are sparse and come predominantly from the recently performed clinical trials [9]. Thus, antazoline’s optimal dosing has not been yet established; instead it is based on clinical experience, and hence varies among different authors [2,3,10,11,12]. In light of the increasing use of antazoline as an AF terminating agent, this gap should be filled. This could also potentially help to understand the mechanisms of the pharmacodynamic activity of the drug. This is particularly important and interesting, as the available data suggest that in human healthy volunteers, the terminal elimination half-life of antazoline was 2.29 h with a mean residence time of 3.45 h. At the same time, the QT/QTcF reported by Piotrowski et al. remains prolonged even 6 h after the last dose, which may suggest a potential role of drug metabolites [1]. Physiology-based pharmacokinetic (PBPK) models allow for dividing the simulated clinical scenario into three separate areas, namely drug/formulation, system (in this case—human body), and trial design [13]. The PBPK models are perfectly positioned to not only test various clinical scenarios (i.e., the potential role of drug-drug interactions for human safety), but also help to formulate scientific hypotheses [14,15]. With that we decided to characterize antazoline’s ADME parameters in vitro, observe its PK in patients, and develop and verify the PBPK model describing antazoline and its main metabolite disposition after intravenous administration.

### 1.2. Study Aims

The study has multiple aims, which are listed below:

1. To perform in vitro experiments towards antazoline’s ADME characterization and present their results,
2. To build and verify the antazoline PBPK model based on the in vitro ADME data with the aim of its pharmacokinetic characterization,
3. To build a model of the antazoline metabolite based on the available phys-chem data and extend the previously built PBPK model.

## 2. Results

### In Vitro Assays

The results of in vitro assays investigating the binding properties and metabolic stability of antazoline are presented in Table 1. Less than 50% of antazoline was bound to plasma proteins. Warfarin (control compound), was bound almost completely as expected, which gives confidence with regard to the method sensitivity and confirms the good quality of the obtained results. Antazoline, in contrast to the control drug, amitriptyline, was only slightly bound to the microsomal membrane. Moreover, antazoline did not bind to erythrocytes. Antazoline’s metabolism was also assessed with the use of hepatocytes and human liver microsomes as in vitro models (Table 1). The cytochrome responsible for metabolism was mainly CYP 2D6. No decrease in antazoline concentration was observed after incubation with any of the glucuronosyltransferases tested. These data are crucial in understanding the drug pharmacokinetics. Together with the QSAR predicted parameters and clinical data they were used to parametrize a PBPK model for antazoline and its metabolite.

For antazoline, the predicted median AUC_inf_ value for a single dose of 100 mg was in good concordance with the observed in vivo in the ELEPHANT I study (825 vs. 910 ng·h /mL). The fold difference (maximal AUC/minimal AUC) was 7.1 and 9.1 for the simulated population and the clinical sample, respectively. The predicted AUC_5h_ for the metabolite was 0.7 fold of the observed. 

**Figure 1:**
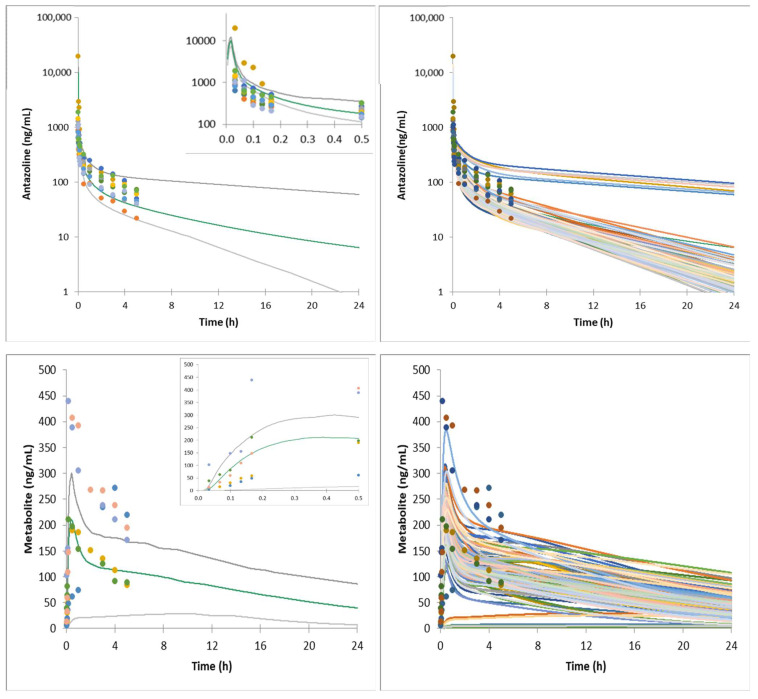
Mean and individual simulated systemic plasma concentration profiles of antazoline and its metabolite following 100 mg of antazoline infusion (healthy volunteers, ELEPHANT I study). Dots—observed individual data; gray lines—5th and 95th percentile; green line—simulated average concentration; colour lines—simulated individual concentrations. Insert—data for the first 30 min after dosing.

The mean and individual simulated plasma concentrations profiles compared with the observed data are presented in Figure 1. The average fold difference between mean predicted (*n* = 100) and mean observed (*n* = 10) antazoline concentration across sampling time points (*n* = 11) was 1.04 (0.66–1.56), with the overestimation within first 10 min after the start of dosing and underestimation after 1 h. The metabolite concentrations were available for five out of 10 volunteers and for eight time points. The metabolite concentration was measured for all five subjects (for 8 time points samples were available for all five subjects, for the remaining three time points, 2, 3, and 4 samples were available). The average fold difference between mean predicted (*n* = 100) and mean observed (*n* = 5) antazoline concentration across sampling time points was 0.82 (0.2 for the first point—1.39).

**Figure 2:**
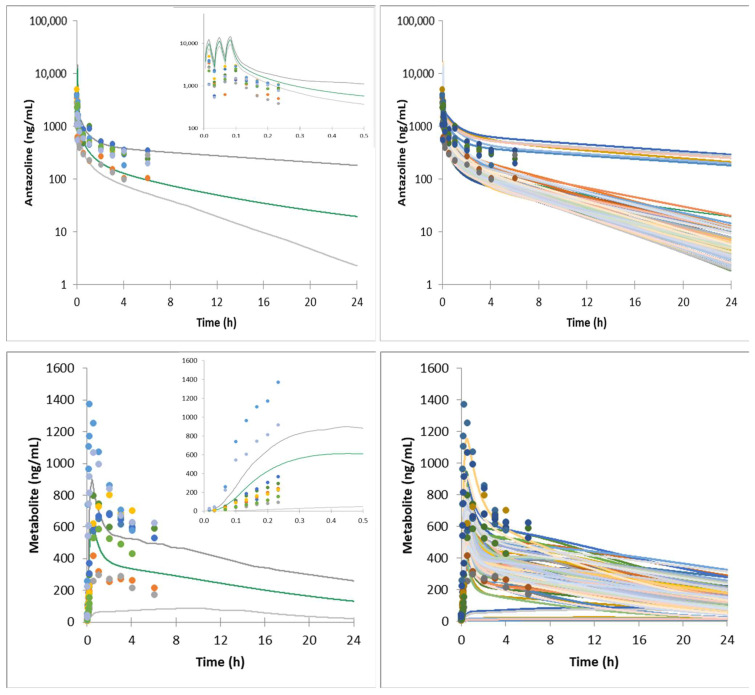
Mean and individual simulated systemic plasma concentration profiles of antazoline and its metabolite following 300 mg antazoline infusion (healthy volunteers, ELEPHANT II study). Dots—observed individual data; gray lines—5th and 95th percentile; green line—simulated average concentration; colour lines—simulated individual concentrations.

**Figure 3:**
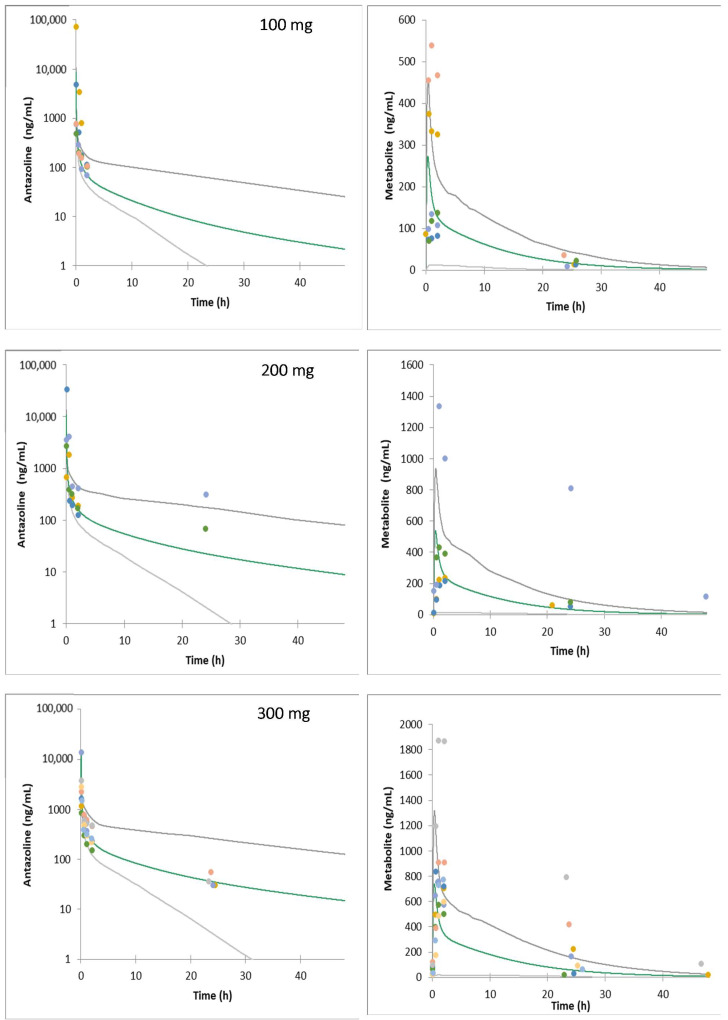
Mean simulated systemic plasma concentrations profiles of antazoline and its metabolite following 100, 200, and 300 mg antazoline infusion (patients, ELEPHANT III study). Dots—observed individual data; gray lines—5th and 95th percentile; green line—simulated average concentration.

The model was verified against ELEPHANT II and ELEPHANT III data (Figure 2 and Figure 3).

## 3. Discussion

In the present work, a PBPK model simulating simultaneously the pharmacokinetic profile of antazoline and its metabolite was developed. The model’s predictive performance was successfully verified against available clinical data. 

The PBPK model building was based on the combination of literature derived and on in house, estimated and measured data. To our knowledge, this is the first study providing in vitro characterization of antazoline’s ADME properties. All of the experiments were run in the standard settings, following current guidelines. As part of the quality control, these experiments were also performed for control substances. The ADME parameters are crucial in understanding the pharmacological properties of the drug. As the aim of the project was to develop a PBPK model with a potential clinical application, it was decided to request running in vitro ADME experiments in external CRO to avoid any potential bias resulting from academic lab-specific settings. 

As the simulation of the formation of the metabolites requires information on individual metabolic pathways, it was decided to use the recombinant CYPs data to describe antazoline’s metabolic clearance. The obtained results of metabolic stability in vitro assays suggest that antazoline is predominantly metabolized by CYP2D6, with a minor role played by CYP2C19. It was assumed that they both lead to the same metabolite formation. There is, however, a difference between the clinically estimated clearance value (80.5 L/h as the result of NCA analysis) and the in vitro—in vivo extrapolated value (70 L/h). This underprediction suggests that the in vitro tested CYP enzymes do not cover all of the clearance mechanisms. For that reason, an additional set of experiments with the recombinant second phase enzymes was run, although there was no activity found for the tested glucuronosyltransferases, namely UGT 1A1, 1A3, 1A4, 1A6, 1A9, 2B7, B15. The lacking value could be the effect of renal or other non-CYP enzymatic metabolism. From the PBPK model’s perspective, the exact mechanism of additional clearance is not crucial, once the below discussed assumption of CYP triggered hydroxy metabolite formation is correct. The CYP mediated clearance was supplemented by the additional systemic clearance of 10 L/h clearance to allow for proper disposition characterization.

For the antazoline metabolite there was no data available to feed the model, therefore several assumptions were made. The clearance value was predicted with the use of pkCSM software, which is based on the analysis of chemical signatures. The predicted value of 12 L/h was assumed to be uncertain to the level which required sensitivity analysis and assessment of its influence on the final results. The same applied for other parameters, namely the phys-chem data. A global sensitivity analysis (GSA) module in a Simcyp Simulator was used, and the Morris method [16] with four levels, 100 trajectories, and one repetition was applied. The tested parameter settings are presented in Table 2.

**Figure 4:**
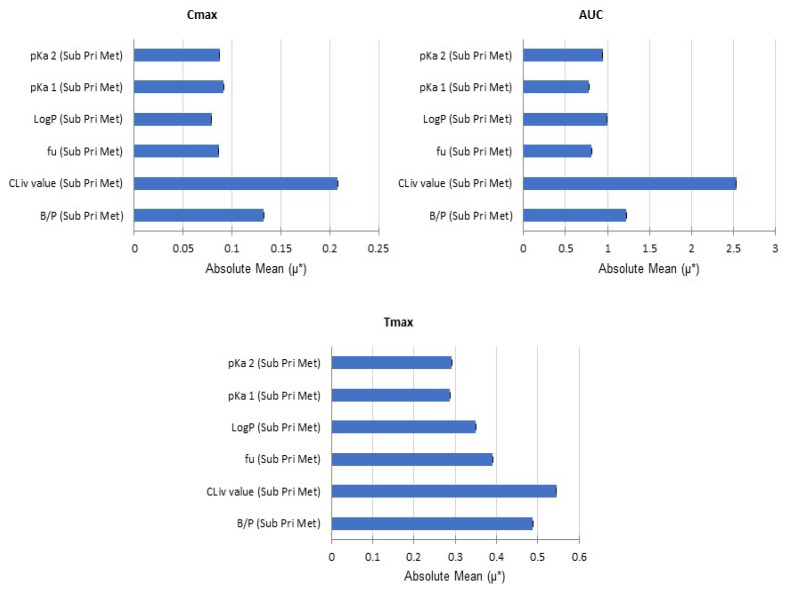
Results of sensitivity analysis for antazoline metabolite model parameters. µ*—the mean of absolute elementary effects of the tested parameter.

The metabolite maximum concentration (C_max_ [ng/mL]), area under the concentration-time curve (AUC0-24 [ng·h/mL]), and time to reach Cmax (T_max_ [h]) were used as the endpoints, and the results are presented below (Figure 4).

The results suggest the significant influence of systemic clearance and to a lesser degree the blood-to-plasma partition ratio.

It is worth noting that significant variability at the level of antazoline and its metabolite concentration in plasma was observed clinically. It can be hypothesized that the reason lies in the genetically encoded CYP2D6 activity, although there was neither a genotyping nor a phenotyping study run to test such a hypothesis. Two individuals participating in the ELEPHANT I study have significantly higher plasma metabolite concentrations, which corresponds with low antazoline concentration (fast metabolism). A similar situation was noted in the ELEPHANT II and ELEPHANT III study results. As shown in Figure 1, Figure 2 and Figure 3, the developed model was able to account for the clinically observed variability, and the simulated individual concentration-time profiles overlay the clinically observed plasma exposure.

The metabolites presence in the body is of potential pharmacological importance as it may, together with its parent antazoline, contribute to its pharmacodynamic effect at the heart’s electrophysiology level. The hypothesis of the potential role of the hydroxy metabolite of antazoline in the cardiological activity of antazoline comes from the analysis of QT/QTcF values reported in the ELEPHANT II study [1]. There was no sharp dQTcF interval increase, and the maximum value of these parameters is reached after 4 h when the antazoline concentration reaches its plateau. Additionally, even after 10 h, the dQTcF interval is still at the top of the plateau, which may suggest the potential role of metabolites in the pharmacodynamic effect formation. This hypothesis requires further investigation and the building of a PK/PBPK-PD model and its simulation results analysis. 

## 4. Materials and Methods

### 4.1. ADME Parameters Analysis

All of the in vitro experiments described below were performed by Cyprotex Discovery Ltd. according to the presented protocols.

#### 4.1.1. Binding Assays

Plasma protein binding: Protein binding was assessed by the rapid equilibrium dialysis (RED) method (*n* = 3). The solution of antazoline mesylate (10 mM) in DMSO was diluted with human plasma (0.5% final DMSO concentration) and placed in the donor chamber of the RED units. The dialysate chamber contained a buffer (pH 7.4). The system was allowed to reach equilibrium at 37 °C. Then, samples were taken from both sides of the semi-permeable membrane and analysed by LC-MS/MS to assess the fraction unbound in plasma (fu). Samples were quantified using standard curves prepared in the equivalent matrix. Cyprotex generic LC-MS/MS conditions were used. Warfarin was used as a control drug.

Blood-to-plasma ratio: The distribution of antazoline mesylate in blood was investigated by incubation with whole blood samples from healthy volunteers (*n* = 3). Antazoline mesylate (final concentration 0.5 µM, final DMSO concentration 0.005%) was incubated with heparinized whole blood, reference red blood cells and reference plasma for 60 min at 37 °C. The whole blood samples were centrifuged for 5 min at 5000× *g* at 4 °C and an aliquot was sampled from the plasma and red blood cell layers for analysis. The lysis of the red blood cells was achieved by three freeze-thawing cycles. The experimental samples and reference samples after protein precipitation (with acetonitrile, 1:2, *v*/*v*), were analysed by LC-MS/MS to assess the ratio of the whole blood concentration vs. the plasma concentration (blood-to-plasma or B:P ratio) with standard deviation. Samples were quantified using standard curves prepared in the equivalent matrix. Methazolamide was used as a control drug. 

Microsomal binding: Solutions of antazoline mesylate (3 µM, 0.5% final DMSO concentration) was prepared in 0.1 M phosphate buffer (pH 7.4), and human microsomes (0.5 mg/mL in 0.1 M phosphate buffer, pH 7.4) were prepared in triplicate. The solutions were added to compartments of an equilibrium dialysis system with a semi-permeable membrane. Incubations were carried out in an atmosphere of 5% CO_2_ with a relative humidity of 95% at 37 °C. After the equilibration, samples were taken from both sides and analysed to assess the fraction unbound in microsomes (fu(mic)). Cyprotex generic LC-MS/MS conditions were used. Amitriptyline was used as a control drug.

#### 4.1.2. Metabolic Stability

Cytochrome P450 Reaction Phenotyping: cDNA expressed human P450 enzyme preparations co-expressed with human NADPH cytochrome P450 reductase (BactosomesTM) were supplied by Cypex Ltd, Dundee, Dundee City, United Kingdom. Antazoline mesylate (1 µM) was incubated with CYP1A2, CYP2C8, CYP2C9, CYP2C19, CYP2D6 and CYP3A4 recombinant isoform expression systems (BactosomesTM). Following pre-incubation of antazoline mesylate with Bactosomes^TM^ (final P450 concentration: CYP1A2 100 pmol/mL, CYP2C8 50 pmol/mL, CYP2C9 25 pmol/mL, CYP2C19 100 pmol/mL, CYP2D6 50 pmol/mL and CYP3A4 25 pmol/mL) in 0.1 M phosphate buffer pH 7.4, NADPH (final concentration 1 mM) was added and compound was incubated at 37° for up to 45 min. Following protein precipitation (with acetonitrile, 1:2, *v*/*v*), and centrifugation at 3000 rpm for 20 min at 4 °C, the sample supernatants were analysed using Cyprotex generic LC-MS/MS conditions. A control compound for each isoform was included in the assay.

UGT Reaction Phenotyping: cDNA expressed human UGT enzyme preparations were supplied by BD Biosciences. Antazoline mesylate (final concentration 5 µM; final DMSO concentration 0.1%) was pre-incubated at 37 °C with UGT enzyme (UGT1A1, UGT1A3, UGT1A4, UGT1A6, UGT1A9, UGT2B7, and UGT2B15) in 50 mM Tris HCl buffer pH 7.5 and alamethicin before the addition of UDGPA (final concentration 2 mM). Antazoline was incubated for up to 60 min with each isoform. Following protein precipitation (with acetonitrile, 1:2, *v*/*v*), the sample supernatants were analysed using Cyprotex generic LC-MS/MS conditions. cDNA expressed human UGT enzyme preparations was supplied by BD Biosciences. A control compound for each isoform was included in the assay.

Microsomal Metabolic Stability: Microsomes were supplied by Corning, Woburn, MA, US. Antazoline mesylate (final concentration 1 µM; final DMSO concentration 0.25%) was pre-incubated with pooled liver microsomes (final protein concentration 0.5 mg/mL), and 0.1 M phosphate buffer pH 7.4 at 37 °C (*n* = 5). Then, NADPH was added to the final concentration of 1 mM, and the solution was incubated for up to 45 min. Following protein precipitation (with acetonitrile, 1:2, *v*/*v*), and centrifugation at 3000 rpm for 20 min at 4 °C, the sample supernatants were analysed by LC-MS/MS (Cyprotex generic conditions). The negative control did not contain the NADPH. Verapamil was used as a control drug.

Hepatocyte stability: HLM were supplied by Bioreclamation IVT, Baltimore, MD, US. Antazoline (final concentration 3 µM; final DMSO concentration 0.25%) was incubated for up to 60 min with a pooled cryopreserved hepatocytes suspension (final cell density 0.5 × 106 viable cells/mL in Williams E media supplemented with 2 mM L glutamine and 25 mM HEPES) at 37 °C (*n* = 6). Following protein precipitation (with acetonitrile, 1:2, *v*/*v*), and centrifugation at 2500 rpm for 30 min at 4 °C, the sample supernatants were analysed by LC-MS/MS (Cyprotex generic conditions). Verapamil was used as a control drug.

#### 4.1.3. Clinical Studies

**Figure 5:**
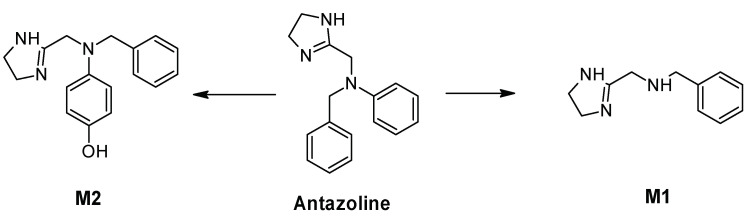
The main pathways of antazoline metabolism.

Metabolite profiling was undertaken using human plasma collected from healthy volunteers receiving a standard intravenous dose of 100 mg of antazoline mesylate [17], and showed two main Phase I metabolites (Figure 5): M1 resulting from the removal of phenyl (N-benzyl-1-(4,5-dihydro-1H-imidazole-2-yl)methenamine), and M2, formed by introducing hydroxyl in the para position into the phenyl of antazoline (hydroxyantazoline). Due to the structural similarity of hydroxyantazoline (M2) to antazoline and its potential antiarrhythmic activity, the characterization of hydroxyantazoline’s concentration in plasma and incorporation into the PBPK model was regarded as crucial. Neither physico-chemical data nor ADME parameters were available for the metabolites, thus QSAR predicted values were used instead (Table 3—drug-specific data).

Sample preparation: 290 µL of plasma and 10 µL of xylometazoline (internal standard, c = 1000 μg/L) was transferred to an Eppendorf tube with 5 mg of mag-MIP. Next, the tube was put on the vortex to provide a contact time with sorbent for 30 min. Then, the supernatant, separated from the sorbent by an external magnetic field, was discarded and the washing step was carried out by applying a volume of 300 μL of ultra-pure water for 0.5 min on the vortex. The supernatant was removed in the same manner as it was described above. Finally, the elution took place by adding a volume of 1000 µL of 40 mM ammonium acetate-methanol, 30:70 *v*/*v* and vortex for 5 min. The eluate was diluted with water pH 3 (1:1 *v*/*v*) and an aliquot of 10 µL was injected into the LC-MS/MS. Quantitative analysis was performed using an Agilent 1260 Infinity system (Agilent Technologies, Santa Clara, CA, USA), equipped with a degasser, an autosampler and a binary pump coupled to a QTRAP 4000 hybrid triple quadrupole/linear ion trap mass spectrometer (AB Sciex, Framingham, MA, USA), as described previously [22]. The turbo ion spray source was operated in positive mode. The curtain gas, ion source gas 1, ion source gas 2 and collision gas (all high purity nitrogen) were set at 345 kPa, 207 kPa, 276 kPa and “high” instrument units (4.6 × 10^−5^ Torr), respectively. The ion spray voltage and source temperature were 5000 V and 600 °C, respectively. The quantitative multiple reaction monitoring transitions, declustering potential (DP) and collision energy (CE) was *m*/*z* 282 > 91 (DP = 96 V, CE = 39 V) for hydroxyantazoline, *m*/*z* 266 > 65 (DP = 81 V, CE = 25 V) for antazoline, *m*/*z* 245 > 145 (DP = 121 V, CE = 63 V) for xylometazoline, and *m*/*z* 261 > 57 (DP = 111 V, CE = 49 V) for oxymetazoline. Chromatographic separation was achieved with a Kinetex^®^ C18 column (100 mm × 4.6 mm, 2.6 µm) from Phenomenex (Torrance, CA, USA). The column was maintained at 40 °C at the flow rate of 0.5 mL min^−1^. The mobile phases consisted of 0.2% formic acid as eluent A and acetonitrile with 0.2% formic acid as eluent B. The gradient (%B) was as follows: 0 min 20%, 1 min 20%, 3 min 95% and 6 min 95%.

Participants: Despite the long market presence of antazoline, no human pharmacokinetic studies had been performed until 2016. The first antazoline clinical trial (ELEPHANT I), conducted by Giebułtowicz et al. [9], assessed plasma concentrations of antazoline mesylate in 10 healthy volunteers following a single intravenous dose of 100 mg (the observed volume of distribution (Vss) was 315 L and the total clearance value was estimated as 80.5 L/h). The second clinical trial with antazoline (ELEPHANT II) aimed at examining its effects on hemodynamic [1]. An ELEPHANT III study assessed both the PK and hemodynamic effects in patients with atrial fibrillation.

The concentration of antazoline and hydroxyantazoline was analysed in the plasma of healthy volunteers from ELEPHANT I (*n* = 5 out of 10 participants, healthy volunteers following a single intravenous dose of 100 mg of antazoline mesylate) [9,17,22], ELEPHANT II (*n* = 10, healthy volunteers, antazoline mesylate was given intravenously in three consecutive boluses (100 mg each) injected over 1 min with 2 min intervals between boluses) [1], and the ELEPHANT III study. The ELEPHANT III study involved patients with atrial fibrillation from the emergency ward of Grochowski hospital. The study protocol was approved by the Local Ethics Committee of The Postgraduate Medical School, Warsaw, Poland (No.). 17/PB/2017 Informed consent was obtained from all individual participants included in the study. All procedures performed in studies involving human participants were in accordance with the ethical standards of the institutional and/or national research committee and with the 1964 Helsinki declaration and its later amendments or comparable ethical standards. Eighteen subjects (12 men and 6 women, mean age 59 ± 14 years) with paroxysmal atrial fibrillation (AF), were enrolled in this study after giving written informed consent. The mean height, weight, and BMI were 1.75 ± 0.09 m, 88 ± 13 kg, and 28.78 ± 4.25 kg/m^2^, respectively. Echocardiographic parameters such as left ventricular ejection fraction was 59 ± 4%. Laboratory parameters were normal in all subjects. Drugs taken by the patients had no liability to interact with CYP 2D6 or CYP 2C. Demographic and clinical characteristics of the studied patients are presented in Table 4. The exclusion criteria were lack of written informed consent, age < 18 years, known allergy to antazoline, the presence of significant heart disease such as heart failure with NYHA class > II, or left ventricular ejection fraction (LVEF) < 40%, uncontrolled hypertension or documented coronary artery disease. Other contraindications included cardiac rhythm other than sinus or systolic blood pressure (sBP) < 90 mmHg.

Antazoline mesylate (Phenazolinum, Polfa, Warsaw, Poland) was administrated intravenously in up to three consecutive boluses of 100 mg each (maximal cumulative dose of 300 mg), injected over 1 min with 2 min intervals between boluses. The drug was stopped immediately after sinus rhythm restoration or when the maximal allowed cumulative dose was given. This drug regimen was chosen based on previous clinical observations, suggesting that to achieve optimal drug efficacy the duration of injections and intervals between boluses should be short and that cumulative doses exceeding 300 mg did not significantly increase drug efficacy. The injection of antazoline could have been stopped immediately if significant side effects occurred. 

Blood samples (c.a. 2 mL) were collected (sodium citrate as an anticoagulant, 3.2%) and the plasma concentration of antazoline and its metabolite were measured before the injection and after 10, 30, 120 min, and after 24 and 48 h. Immediately after collection, blood samples were centrifuged at 2000× *g* for 15 min at room temperature, and plasma samples were aliquoted and stored at −80 °C until analysis.

### 4.2. PBPK Modelling and Simulation—Development and Qualification

#### Models

A set of data on physico-chemical parameters, in vitro metabolic stability and blood binding studies results, and concentration-time profiles from clinical study with antazoline was used for the PBPK model development and verification [9]. Model building was done with the use of the Simcyp Simulator V19 (Certara, Sheffield, UK) [13]. Simcyp compound files were newly developed for both antazoline and its metabolite based on the parameters obtained from newly generated in vitro data presented in this paper, derived from scientific literature, and/or predicted by relevant QSAR models. A full-PBPK model of distribution with perfusion-limited organ models was applied for all tissues, and Method 2, based on Rodgers and Rowland’s model [23], was used to predict the volume of distribution at a steady-state for the parent compound and the metabolite. The predicted Vss was similar to the value reported by Giebułtowicz et al. [9] (4.4 L/kg). Intrinsic clearance values estimated in a recombinant CYP expression system (Bactosomes^TM^) allowed us to identify the CYP2D6 and CYP2C19 enzymes as those involved in the antazoline metabolism. Enzyme specific kinetic data were used to enable metabolite formation. Clearance obtained with the in vitro model was scaled to human in vivo situations using inter-system extrapolation *factors* (ISEFs) specific for Bactosomes^TM^ [24]. Antazoline clearance via the CYP2D6 pathway was equally split into two metabolic products, which was supported by the in vivo data [17]. The body clearance of antazoline triggered by with CYP2D6 and CYP2C19 enzymes was lower than the clearance estimated based on the data from the clinical study. Furthermore, in vitro and in vivo studies indicated that other CYPs can be involved in the elimination of antazoline to some extent, and some other metabolites are also formed. Therefore, an additional systemic clearance of 10 L/h was added to the model. The additional clearance value was calculated as the difference between total body clearance estimated clinically and clearance scaled from in vitro experiments, as presented above.

For the antazoline metabolite, neither blood binding data, Vss, nor clearance was reported in the literature, thus the fraction unbound in plasma, the blood-to-plasma ratio, and the volume of distribution values were predicted with the relevant QSAR models built-in to Simcyp simulator. Total clearance value was predicted using pkCSM platform (http://biosig.unimelb.edu.au/pkcsm/, accessed on 15 October 2020) [21]. The drug-specific input parameters are presented in Table 4 (drug-specific data).

The design of virtual trials followed those reported for the clinical trials concerning demographics, dose, and dosing regimen. A Simcyp NEurCaucasian population was used for the simulations during model development and verification. The developed PBPK model was verified against the PK results of the ELEPHANT II and ELEPHANT III clinical studies. Area under the concentration-time curve (AUC) was used as the comparator. The results were also analysed based on the visual check.

The Simcyp input parameters for the final PBPK model are detailed in Table 4.

## 5. Conclusions

To conclude, antazoline is a clinically useful drug used for the termination of paroxysmal atrial fibrillation, but the detailed mechanism of action is not known. In this study, ADME parameters of antazoline were characterized in vitro and a whole-body PBPK model was developed to predict antazoline pharmacokinetics. The model accounts for the clinically observed variability at the level of plasma concentration and can be used to predict antazoline PK profiles for various dosing regiments, in different populations, or in case of CYP2D6- or CYP2C19-mediated drug interactions.

## Figures and Tables

**Table 1:** Antazoline in vitro blood binding and metabolism data.

**Binding Assays**				
**Parameter**	**Compound**	**Mean**	**SD**	
Blood to Plasma Ratio (B/P)	Antazoline	1.18	0.0161	
	Methazolamide (C)	10.9	3.81	
Fraction Unbound (fu)	Antazoline	0.586	0.0109	
	Warfarin (C)	0.016	0.0002	
Microsomal Binding (fu)	Antazoline	0.88	0.00563	
	Amitriptyline (C)	0.47	0.00381	
**Test System**	**Compound**	**CL_int_** **[µL/min/10^6^ cells]**	**SE CL_int_**	**t_1/2_** **[min]**
Hepatocytes	Antazoline	21.1	2.21	65.8
	Verapamil (C)	78.6	3.85	17.6
HLM	Antazoline	97.4	8.63	14.2
	Verapamil (C)	373	23.7	3.72
**Recombinant CYP Isoform**	**Compound**	**t_1/2_** **[min]**	**SE t_1/2_**	**Bactosomes™ Final P450 Concentration** **[pmol/L]**
2D6	Antazoline	0.56	0	50
	Dextromethorphan (C)	1.53	1.17	50
2C9	Antazoline	-	-	25
	Diclofenac (C)	1.91	0.0800	25
2C19	Antazoline	51	2.84	100
	Omeprazole (C)	1.53	0.420	100
3A4	Antazoline	-	-	25
	Testosterone (C)	24.6	4.12	25
1A2	Antazoline			100
	Ethoxycoumarin (C)	2.23	1.00	100
2C8	Antazoline	1100	4210	50
	Paclitaxel (C)	13.4	0.880	50
**UGT Isoform**	**Test Concentration**	**Recombinant UGT CL_int_ Clearance** **[µL/min/mg]**	**Recombinant UGT Half-Life t_1/2_** **[min]**	
1A1	1 µM	<2.9	>240	
1A3	1 µM	<2.9	>240	
1A4	1 µM	<2.9	>240	
1A6	1 µM	<5.8	>240	
1A9	1 µM	<5.8	>240	
2B7	1 µM	<5.8	>240	
B15	1 µM	<5.8	>240	

**Table 2:** Sensitivity analysis setup for antazoline metabolite model parameters.

Parameter	Distribution	Lower Bound	Upper Bound
LogPo:w Value	Uniform	1.5	3.5
pKa 1 = acid Value (Ampholytes)	Uniform	6	14
pKa 2 = base Value (Ampholytes)	Uniform	6	14
Blood to plasma partition ratio	Uniform	0.55	2
i.v. Clearance value	Uniform	2	80
Fraction unbound in plasma	Uniform	0.1	1

**Table 3:** Simcyp input parameters for final antazoline PBPK model.

**ANTAZOLINE**			
Phys-chem Parameters	Compound type	Monoprotic Base	Source
	MW	265.35 g/mol	PubChem
	logP	3.16	Average of PubChem; ALGPS; ChemAxon reported values
	pKa	9.43	Average of PubChem; ChemAxon, Toxnet [18,19,20]
Blood binding	fu plasma	0.586	In house data/Cyprotex
	B/P	1.18	In house data
Distribution	Vss	4.97	Simcyp Method 2
Elimination	CLint HLM	97.4 (SE 8.63) µL/min/mg protein	In house data
	CLint hepatcyte	21.1 (SE 2.21) µL/min/mg protein	In house data
	CLint CYP2D6	24.75 µL/min/pmol	In house data, Bactosomes
	CLint CYP2C19	0.14 µL/min/pmol	In house data, Bactosomes
	Additional systemic clearance	10 L/h	Estimated *
**M2 METABOLITE**			
Phys-chem parameters	Compound type	Ampholyte	
	MW	281.36 g/mol	PubChem
	logP	2.57	ChemAxon
	pKa1	9.2	ChemAxon
	pKa2	10.3	ChemAxon
Blood binding	fu plasma	0.742	Simcyp QSAR model
	B/P	1	Assumed
Distribution	Vss	2.17	Simcyp Method 2
Elimination	CL total	12 L/h	pkCSM [21]

* According to the clinical reports, total systemic clearance equals 80.5 L/h; however, clearance scaled from an in vitro CYPs experiment gave only 70 L/h, thus 10 L/h was added as an additional systemic clearance to account for the gap.

**Table 4:** Demographic and clinical characteristics of patients participating in the ELEPHANT III study.

N = 18	Mean	SD
**Age [Years]**	59	14
Gender (M/F)	12/6	
BMI [kg (m^2^)^−1^]	28.8	4.25
**Echocardiographic Parameters** LVEF (%)	59	4
**Laboratory Tests**		
WBC level [K/µL]	6.8	1.9
HGB level [g L^−1^]	14.6	1.4
HCT %	42.7	3.7
Platelet Count (PLT) [K/µL]	220	51
Creatinine level [mg L^−1^]	1.04	0.38
Sodium level [mmol L^−1^]	141	2
Potassium level [mmol L^−1^]	4.3	0.2
**Concomitant Disorders**	N (%)	
Atrial fibrillation	18 (100%)	
Hypertension	14 (78%)	
Hyperlipidemia	6 (33%)	
Coronary arterial disease	1 (5.5%)	
Chronic heart failure	1 (5.5%)	
Chronic obstructive pulmonary disease	1 (5.5%)	
Diabetes mellitus	2 (11%)	
Obstructive sleep apnea	2 (11%)	
Hypothyroidism	4 (22%)	
Renal failure	1 (5.5%)	
**Antiarrhythmic Drugs**		
Beta-blocker	11 (61%)	
Propafenon	9 (50%)	
Sotalol	3 (17%)	
Amiodaron	1 (5.5%)	
None	1 (5.5%)	

M/F—male/female; BMI—body mass index; LVEF—left ventricle ejection fraction; WBC—white blood cell count; HGB—hemoglobin; HTC—hematocrit; SD—standard deviation.

## Data Availability

Data is contained within the article.
